# Effect of cusp coverage and water storage on compressive strength of composite restorations of premolars

**DOI:** 10.4317/jced.54668

**Published:** 2018-04-01

**Authors:** Shila Emamieh, Parvaneh Hojati, Amir Ghasemi, Hasan Torabzadeh

**Affiliations:** 1 Assistant Professor, Department of Restorative Dentistry, School of Dentistry, Shahid Beheshti University of Medical Sciences, Tehran, Iran; 2 General Dentist, Private practice, Tehran, Iran; 3 Professor, Department of Restorative Dentistry, School of Dentistry, Shahid Beheshti University of Medical Sciences, Tehran, Iran; 4 Professor, Iranian Center for Endodontics Research, Shahid Beheshti University of Medical Sciences, Velenjak, Chamran Highway, Tehran, Iran

## Abstract

**Background:**

The aim of this study was to assess the effect of cusp coverage and water storage on compressive strength of composite restorations.

**Material and Methods:**

This *in vitro* experimental study was conducted on 40 extracted human maxillary premolar teeth, which were randomly divided into four groups of 10. Mesio-occluso-distal (MOD) cavities were prepared in all teeth. The thickness of composite for cusp coverage was 1.5 mm in groups 1 and 3 and 2.5 mm in groups 2 and 4. Compressive strength (CS) was measured after 24 hours in groups 1 and 2 and after six months of water storage in groups 3 and 4. Two-way ANOVA was used to statistically analyze the data.

**Results:**

The mean and standard error (SE) of compressive strength was 795.23 ± 35.18N in Group 1, 1232.52 ± 78.01N in Group 2, 617.18 ± 40.19N in Group 3 and 963.22 ± 50.05N in Group 4.

**Conclusions:**

Statistical analysis showed a significant difference in compressive strength measured after 24 hours (groups 1 and 3) and after six months of water storage (groups 2 and 4). The compressive strength of groups with 2.5 mm cusp coverage was significantly greater than that of groups with 1.5 mm cusp coverage.

** Key words:**Fracture strength, cusp coverage, water absorption, composite resin, compressive strength.

## Introduction

Cusp coverage is a suitable modality for reinforcement of severely damaged teeth with weakened cusps due to extensive caries or iatrogenic removal of tooth structure. The wide range of cusp coverage has been proposed for strengthening of weakened teeth is 1.5-2.5 mm. In such cases, cusp coverage is preferred to a simple intra-coronal restoration (1). Cusp coverage, although ideal in specific cases, has some drawbacks. For instance, it requires high level of proficiency and skills of the operator and in the esthetic zone, non-tooth-colored restorative materials is not often accepted by patients (2).

Water sorption in composites follows the diffusion law. Due to the presence of ions and several compounds in the saliva, the osmotic gradient from the material towards the saliva decreases. Thus, the release of unreacted ions and monomers in the composite structure into the saliva decreases as well. Moreover, as the result of reduced osmotic gradient, diffusion of water molecules from the saliva into the polymer network of composite decreases. Resultantly, the solubility, water sorption and expansion of composite decrease. Another important issue is that the saliva components such as enzymes, acidic and alkaline substances, bacteria and their byproducts can cause superficial degradation of composite and increase its solubility and decrease its mechanical properties (3).

The aim of this study was to assess the effect of cusp coverage and water storage on compressive strength of composite restorations.

## Material and Methods

This study was conducted on 40 sound human maxillary premolar teeth extracted due to periodontal or orthodontic reasons. All teeth were inspected under a stereomicroscope at ×10 magnification to ensure absence of cracks and caries on root and crown surfaces. The teeth were immersed in 0.5% thymol for 24 hours in order to be disinfected.

To minimize the influence of size and shape variations on the results, the teeth were classified according to their mesiodistal and buccolingual dimensions. The teeth were randomly divided into four groups of 10. In groups 1 and 2, cusp coverage was performed with 1.5 and 2.5mm thickness of composite resin, respectively and compressive strength was measured 24 hours after restoration. In groups 3 and 4 cusp coverage was performed with 1.5, 2.5mm thickness of composite resin, respectively and compressive strength was measured after six months of water storage. The teeth were mounted in acrylic resin up to the level of the cementoenamel junction. A putty impression (Speedex; Coltène/Whaledent Inc., Cuyahoga Falls, OH, USA) was taken from the tooth crown to the level of cementoenamel junction. The prepared silicon mold was buccolingually sectioned in half by a scalpel.

**Figure 1:**
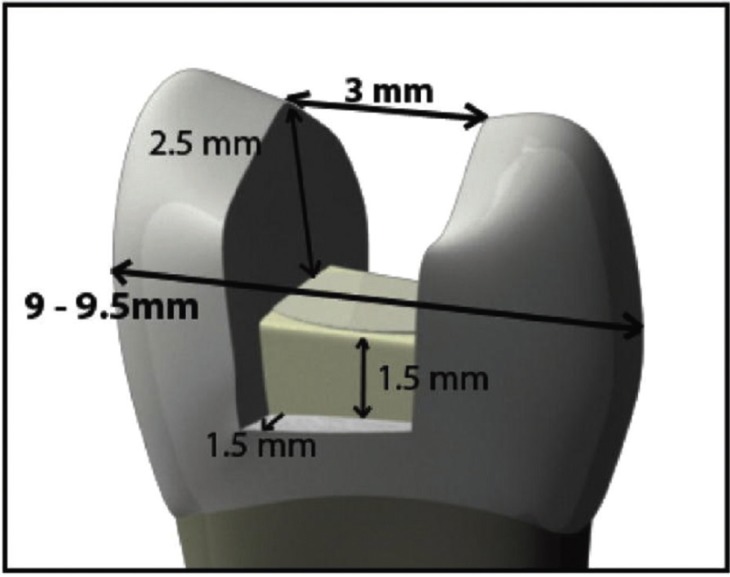
The dimensions of the cavity.

**Figure 2:**
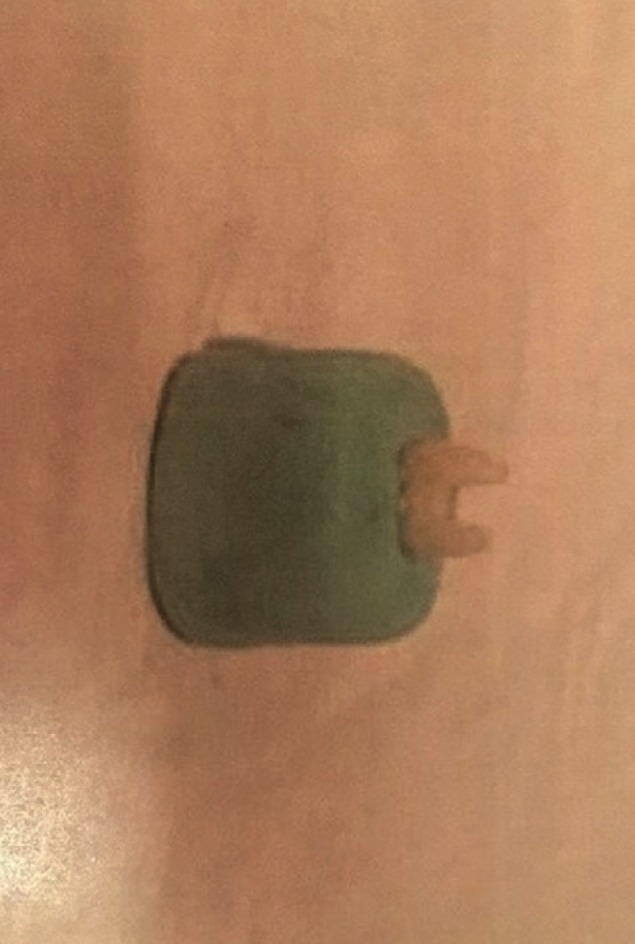
Samples after cusp reduction.

A MOD cavity was prepared with an isthmus width equal to two/thirds of the distance between the two cusp tips (Fig. 1). The buccal and palatal cavity wall was parallel to its corresponding wall having 3mm depth. The distance between the box floor and cementoenamel junction was 1mm. After creating 3 grooves as a guide, buccal and palatal cusps were reduced using a short fissure bur while maintaining the cusp slope (Fig. 2). After cavity preparation and irrigation, the teeth were etched according to total etch technique; 37% phosphoric acid (Ultra-Etch, Ultradent Products Inc, South Jordan, UT, USA) was applied on the enamel and after five seconds on dentin for 15 seconds followed by rinsing for 10 seconds and drying excess water using a cotton pellet. Two consecutive coat of Single Bond 2 (3M Dental Products, St. Paul MN 55144, USA) was applied on etched surfaces for 15 seconds with gentle agitation using a fully saturation applicator and gently air thinned for five seconds to evaporate solvents and light cured for 20 seconds using a QTH light-curing unit (Blue Point, Arialux, Tehran, Iran) with a light intensity of 705mW/cm2. Z250 composite (3M ESPE, St. Paul, MN, USA) was incrementally applied into the cavity (increment thickness of <2mm) and each increment was light-cured for 40 seconds. For application of final layer (occlusal area), Z250 composite was applied into the silicon mold and the tooth was placed inside the mold. Excess composite was removed and the final layer was light-cured for 40 seconds. The composite was finished and polished and the samples were stored in distilled water at 37°C for 24 hours and 6 months until testing.

Samples were subjected to stress in a universal testing machine (Zwick Roell, Ulm, Germany). Load-applying compartment was semi-circular with 3.7mm diameter, which contacted both buccal and palatal cusp slopes and applied load to halfway the distance between the cusp tip and central groove. Load was applied on the occlusal surface of the samples at a crosshead speed of 1mm/minute. Load was increased until fracture occurred. Maximum load in Newton, which caused fracture, was recorded. Two-way ANOVA was used to compare compressive strength of the groups.

## Results

The results are shown in  Table 1. Data had normal distribution in all four groups. Two-way ANOVA was applied to compare compressive strength in terms of thickness of composite for cusp coverage and duration of water storage. The results showed that cusp coverage by 2.5mm yielded significantly higher compressive strength than 1.5mm (*P*<0.001). Comparison of the two time points showed that the compressive strength was significantly higher in immediate groups compared to those stored in water for six months (*P*<0.001). The interaction effect of the two factors was not significant (*P*=0.399).

**Table 1:**
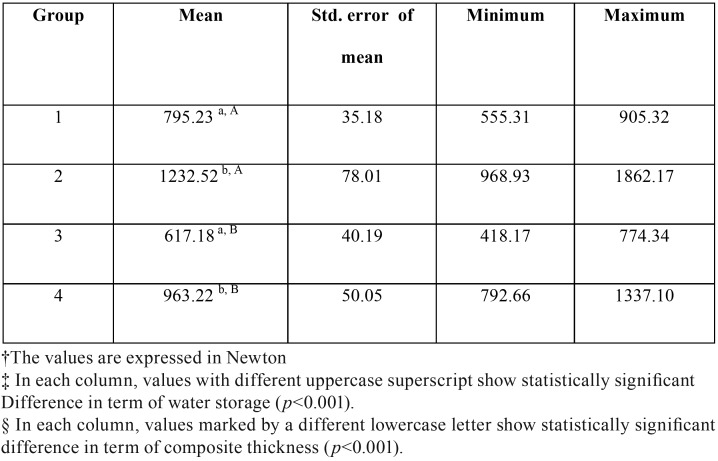
The mean compressive strength† of four groups‡, §.

## Discussion

Evidence shows high success rate of properly bonded cusp coverage restorations due to advances in properties of composite resins (4). Improved composite properties led to a reduction in use of amalgam and increased the demand for direct composite restorations instead of indirect onlays or full coverage crowns (5). The current study aimed to assess the effect of two important factors namely: thickness of composite for cusp coverage and water storage on compressive strength of composite restorations.

Premolar teeth were used in this study because sound premolars were easier to find than other teeth and many orthodontic treatment plans require the extraction of premolar teeth (6). Also, most previous studies were conducted on premolars and thus, comparison of results could be done more efficiently. Moreover, the possibility of clinical fracture of premolar teeth is high due to their particular morphology and sharp cusp slope, resulting in separation and fracture of cusps during mastication (7). Linn reported high prevalence of fracture in maxillary second premolars with MOD restorations (8). Also, premolars are often visible in a full smile and when speaking; thus, tooth-colored restorative materials are often required for restoration of premolars (9). Thus, direct composite restorations were used in the current study, since they provide optimal esthetics and can be performed within one session with fraction of a cost of an indirect restoration.

Since shape, form and size of teeth affect tooth fracture, in the current study the teeth were classified based on their crown height and buccolingual width, because these values dictate the dimensions of the MOD cavity to be prepared and are among the most important factors affecting fracture strength (10). It appears that cavity preparation is mainly responsible for loss of stiffness and consequent fracture of restoration (11).

Georges *et al.*, in 2003 reported cavity depth to be the most critical parameter in cavity preparation affecting fracture strength. They added that as little as 1mm increase in cavity depth significantly decreased the fracture strength of teeth (12). Thus, cavity depth was 3mm in the current study that was one of the weakest tooth condition.

In general, fracture strength of a tooth with a MOD cavity is 50% lower than that of sound teeth (3). Also, 39-61% reduction in stiffness following a MOD cavity preparation has been reported (13). Thus, MOD cavities were prepared in the current study.

Another important factor in preparation of MOD cavities is the depth and width of the cavity and dentin thickness between the axial walls. In the current study, the isthmus width of the MOD cavity in premolars was two-thirds of the distance between the two cusp tips to justify cusp coverage. Cusp coverage is indicated when the cavity width exceeds half the distance between the two cusp tips; cusp coverage is necessary if this width exceeds two-thirds of the distance. Bell *et al.*, in 1982 emphasized that cusp coverage improved the prognosis of teeth in long-term (14). Thus, cups coverage is preferred to intra-coronal restorations in severely damaged teeth 1. According to a study by Panahandeh *et al.*, in 2015 the greatest stress distribution occurred in teeth with 2.5mm cusp coverage. They reported the compressive strength of teeth with 1.5mm cusp coverage to be equal to that of sound teeth (15). Thus, we assessed the effect of 1.5 and 2.5mm thickness of composite for cusp coverage on compressive strength of restorations.

The current study revealed that 2.5mm cusp coverage provided significantly higher compressive strength than 1.5mm cusp coverage. Mondelli *et al.*, in 2002 showed that 2mm cusp reduction and its direct restoration with composite resin reinstated the fracture strength of weakened teeth (16). Barreto *et al.*, in 2015 reported that weakening of cusps had no effect on cusp deflection (deformation) but decreased fracture strength 17. In contrast, Torabzadeh and Sanei in 2014 found no significant difference in fracture strength of groups with 1.5 and 2.5mm cusp coverage, which was in contrast to our findings (6).

Lina *et al.*, and Soares *et al.*, in 2008 stated that cusp coverage decreased fracture strength of teeth (18,19), which was in contrast to our results. Such a controversy in the results of studies may be attributed to the fact that in the aforementioned studies, approximately two-thirds of the occlusogingival height was removed for cusp coverage, and such a great reduction of tooth structure was the main reason behind significant reduction of fracture strength of teeth (and not the coverage of cusps).

Irrespective of the differences in shape of filler particles, resin components and diversity of solvents, previous studies have reported controversial results regarding the effect of moderate- and long-term water storage on mechanical properties of dental composites (20). Llie *et al.* in their study in 2009 reported that storage conditions and type of composite were the most influential factors affecting the macro-mechanical properties (strength and modulus of elasticity) of composites (21). Considering the gap of information on the effect of long-term water storage on fracture strength of teeth with cusp coverage, this study aimed to assess the compressive strength of composite restorations after water storage for six months.

The current study showed that after 24 hours, compressive strength was higher than that of six months of water storage. Kildal *et al.*, in 1994 and Drummon *et al.*, in 1998 discussed that water storage did not affect the volume of materials, and hydrolytic degradation was limited to their superficial layer (22,23), which was in contrast to our findings. Palin *et al.*, in 2005 found that increasing the duration of water storage did not cause a reduction in compressive strength of tested materials. They reported that increasing the duration of water storage did not cause a reduction in compressive strength of tested materials (24).

Some studies have shown a significant reduction in three-point flexural strength of composites following six months of water storage (26 weeks); they showed significant water sorption in different composites, which had a degrading effect on their resin matrix; this can adversely affect the filler-resin bond and cause a reduction in flexural strength (20).

The current results showed that water storage decreased the compressive strength of composite restorations, which was in agreement with the results of Ferracane *et al.*, in 1998. They showed that long-term water storage of methacrylate-based composites affected their mechanical properties. In another study, water storage of composites, irrespective of their type, significantly decreased their fracture toughness in the first six months but the changes were not significant thereafter up to two years 20. Alijabo *et al.*, in 2015 discussed that water sorption of composite samples linearly increased with time and caused a reduction in strength (25).

It appears that water storage causes disintegration of dental composite and results in loss of filler particles (26), destruction of polymer matrix (27) and disintegration of filler-matrix bond (28). Evidence shows that long-term water storage may have variable effects on mechanical properties of materials. For instance, irrespective of the type of composite, water storage can decrease fracture toughness but has less significant effects on other mechanical properties such as flexural strength, modulus of elasticity and hardness, which indicate limited decomposition of composite in water (20). Lassila *et al.* demonstrated that water storage caused a permanent reduction in mechanical properties of polymer matrix, which was in line with our findings (29). The current study aimed to assess the effect of two important factors namely: thickness of composite for cusp coverage and water storage on compressive strength of composite restorations. Comparison of fracture strength of restorations with that of sound teeth was out of the scope of this study. Moreover, some previous studies on compressive strength of sound teeth reported value, which were similar to the value in the group with 2.5 mm cusp coverage in our study (30). Therefore, there was no need to use sound premolar teeth as a control group in our study.

## Conclusions

Thickness of composite for cusp coverage and duration of water storage affect the fracture strength of restored teeth. Cusp coverage with 2.5mm thickness of composite further increased the fracture strength of teeth compared to 1.5mm thickness. Water storage for six months caused a reduction in compressive strength of composite restorations, but no significant difference was noted in this respect between the two groups with 1.5 and 2.5mm thickness of cusp coverage.
